# Cytokine Indexes in Pemphigus Vulgaris: Perception of Its Immunpathogenesis and Hopes for Non-Steroidal Treatment

**Published:** 2017

**Authors:** Mohsen Masjedi, Nafiseh Esmaeil, Ali Saffaei, Bahareh Abtahi-Naeini, Mohsen Pourazizi, Shaghayegh Haghjooy Javanmard, Ali Asilian

**Affiliations:** a *Department of Immunology, Isfahan University of Medical Science, Isfahan, Iran. *; b *Pharmacy Students’ Research Committee, School of Pharmacy, Isfahan University of Medical sciences, Isfahan, Iran. *; c *Skin Diseases and Leishmaniasis Research Center, Department of Dermatology, Isfahan University of Medical Sciences, Isfahan, Iran. *; d *Cancer Research Center, Semnan University of Medical Sciences, Semnan, Iran. *; e *Department of Physiology, Applied Physiology Research Center, Isfahan University of Medical Sciences, Isfahan, Iran. *; f *Department of Dermatology, Isfahan University of Medical Science, Isfahan, Iran.*

**Keywords:** Pemphigus vulgaris, Th1 cytokines, Th2 cytokines, Interleukin, Interferon

## Abstract

Pemphigus vulgaris (PV) is a chronic autoimmune blistering disease of the skin, in which loss of adhesion between keratinocytes is caused by autoantibodies. It has been hypothesized that cytokines play an essential role in the pathogenesis of PV. This study aimed to investigate the other immunopathological aspects of PV by determining the serum levels of cytokines in PV patients to find another treatment strategy except corticosteroid therapy.

Twenty-three patients with PV and a control group consisting of 24 healthy subjects were studied. Interleukin (IL)-2, IL-4, IL-6, IL10, IL-12, IL-17 and interferon-gamma (IFN-γ) were measured in the sera of patients by the enzyme-linked immunosorbent assay (ELISA) method.

The serum levels of IL-2, IL-4, IL-17 and IFN-γ in most patients and controls were undetectable. The serum concentrations of IL-10 in the patients and controls were undetectable, nevertheless, the mean serum levels of this cytokine was 64.375 pg/mL in four patients. The mean serum levels of pro-inflammatory cytokine IL-6 increased significantly in the patients, compared to the controls (169.50 vs. 75.62 pg/mL) (P < 0.05). The same was observed for another pro-inflammatory cytokine, IL-12 (135.33 vs. 86.28 pg/mL) (P < 0.05).

Based on the results of this study it can be concluded that the Type 2 T helper cytokine (IL-6) and macrophage-derived cytokine (IL-12) have essential roles in PV pathophysiology. In addition, the potential clinical application of Th1/Th2 type cytokine-based therapy in PV should be considered in next studies.

## Introduction

Pemphigus vulgaris (PV) is a chronic autoimmune blistering disease of skin. In this disease, loss of adhesion between keratinocytes is induced by autoantibody production, which is associated with both Th1 and Th2 (1, 2). Many anti-cytokine therapies were developed as important options in treating autoimmune diseases. Recent reports suggested notable effects of biologics resulting in dramatic changes in treatment algorithms of several inflammatory diseases including Crohn’s disease and rheumatoid arthritis. Many of these anti-cytokine therapies are now confirmed as safe and effective options in various autoimmune diseases, nevertheless there are very limited clinical reports with these options in PV. Hence, additional clinical studies utilizing these drugs in PV patients are necessary. Moreover, even though additional cytokines are discovered to play important roles in the development of PV, biologics can be developed against these molecules and developed as potential therapeutic strategies (3, 4). Understanding the cytokines profile pattern of PV help to find the pathogenesis of PV and possibly management of this fatal disease (4). 

Cluster of differentiation CD4^+^ T-cells either Th1 or Th2 cells are commonly responsible in the disease eradication or expansion. Th1 cells usually create a large quantity of interleukin-2 (IL-2) and interferon-γ (IFN-γ) which are vital for macrophage activation leading to enhancement of microbial killing and function under cell mediated immunity to destroy the intracellular viruses and tumor cells. Conversely, Th2 cells create IL-4, IL-6 and IL-10, which are involved in the stimulation of the humoral arm of the immune response, activating and preparing B cells for antibody production involved with the extracellular pathogens and allergic responses and they are directly involved in the loss of adhesion between the keratinocytes (5-7). Recent studies suggest that PV pathogenesis is the result of autoantibody production due to an imbalance in the Dsg3-sensitized. Th1 and Th2 cell pathways and Th2 predominant response in PV (3, 4).

IL-6 is a pro-inflammatory cytokine, which is involved in the regulation of the immune cells, including the onset and resolution of inflammation, responses to infection, tissue remodelling and cancer. The pathogenesis of autoimmune diseases is mostly characterized by pro-inflammatory cytokine production. Therefore, these molecules have become the focus of many anti-cytokine therapeutic strategies (8, 9).

IL-12 is another cytokine that plays an important role in autoimmunity diseases. The key Th1 mediators are IFN-γ and IL-12 which positively feedback and stimulate the further differentiation of Th1 cells (3, 10).

IL-17-generating T lymphocytes have newly been categorized as a novel effector T lymphocyte subset, termed Th17 that is discrete from Th1, Th2 and Treg subsets (4, 11). IL-17 is a CD4+ T cell-originated cytokine, which stimulates the production and expression of pro-inflammatory cytokines, IL-beta and TNF-alpha, by the human macrophages and dysregulation of this new subset has been shown to be associated with many autoimmune diseases (12). As mentioned above, the cytokines play a significant role in the immunopathogenesis of PV (3), consequently their measutrement in the serum contribute into the improvement of our own knowledge concerning the immunopathogenesis of this potentially fatal disease (13). Also Rituximab is an anti CD 20 monoclonal antibody that targets the CD-20 molecule found on the cell surface of B-cells. Rituximab has been used in different autoimmune diseases such as PV with using different protocols. This treatment strategy lead to targeting and destroying all B cells. Concequently, the adverse effects of this drug is undeniable.On the other hand, with detecting the responsible cytokines in PV patogenesis, the biological agents can be developed against these cytokines as potential therapeutic strategies (3, 4 and 14). Hence, these therapeutic strategies are more specific and less adverse effects are expected due to their specificity. 

The aim of this study was to determin the cytokine levels in the PV patients in comparison with healthy subjects.

## Experimental

In this case-control study, the sample collection was carried out to evaluate the effects of Th1 and Th2 cytokines in the pathogenesis and severity of PV. The Ethics Committee of Isfahan University of Medical Sciences, Isfahan, Iran, approved this study. The serum samples from 23 age-matched patients with PV and 24 healthy subjects (without any complication such as, septicaemia, metabolic disturbance, temperature deregulation) devoid of any immunosuppressive therapy such as, systemic steroids, cyclophosphamide or azathioprine in the last one month were studied. The diagnosis of PV patients was verified by histopathology (H&E staining method) and direct immunofluorescences, who were admitted to the Department of Dermatology of the Al-Zahra Hospital, Isfahan, Iran, between the years of 2004 to 2014 and all the selected patients had mild to severe diseases, based on the Mahajan’s scoring system (15). Blood samples of 24 healthy subjects, who gave consent for this study, were collected from different departments of Isfahan University of Medical Sciences. Detailed patient history was taken and a physical examination performed. 

**Table1:** Demographic features of PV patients.

Age	±
**Sex ratio (F:M)**	14:9
**Lesions**	60.8% Localized 39.1% systemic
**Geographic dependence**	%69.5 from Isfahan city; %30.4 from Isfahan province
**Recurrence**	8.6% relapsed 91.3%; newly diagnosed
**Family history**	Negative

**Table2:** Level of serum Cytokines in the PV patients and controls

**Cytokines**		**Patients**	**Controls**	**Normal Range**	**P**
Th1	IL-2 IL-12 IFNγ	UD 135.33 UD	UD 86.28 UD	<2.5 (41.10-97.82) <3.0	NS S NS
Th2	IL-4 IL-6 IL-10	UD 169.50 UD	UD 75.62 UD	<4.1 (36.25-149.96) <1.5	NS S NS
Th17	IL-17	UD	UD	<0.5	NS

UD: Undetectable

NS: Not Significant

**Figure 1:**
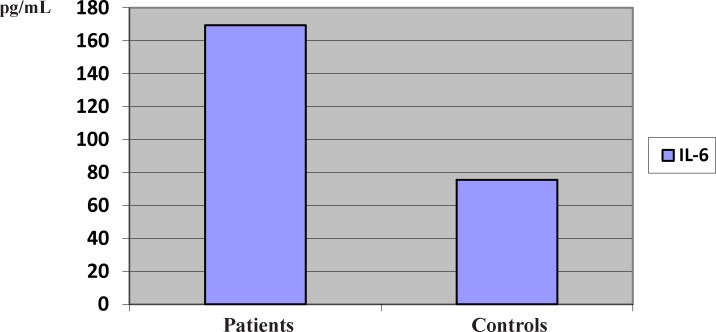
The serum levels of IL-6 in the patients with pemphigus vulgaris

**Figure2:**
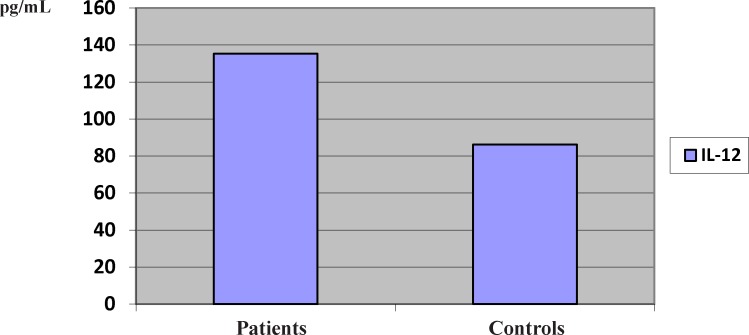
The serum levels of IL-12 in the patients with pemphigus vulgaris.

Patients were diagnosed and managed as per hospital protocol. Ten millilitres of venous blood was taken from all subjects and drawn into tubes free of endotoxins. The tubes were centrifuged at 3000 rpm for 10 min (Sigma 301, US) and then serum was separated and kept at -70 °C . The levels of all markers in the sera were measured by the high sensitivity of the ELISA method. The ELISA kit for the measurement of serum levels of IFN-γ, IL-2, IL-4, IL-6, IL10 and IL-12 was supplied by the U-CyTech biosciences, Yalelaan 48, 3584 CM Utrecht, and the Netherlands. However, the ELISA kit for the measurement of the serum level of IL-17A was supplied by the European Headquarters, Peprotec EC. London. 

*ELISA method for the measurement of Th1 cytokines (IL-2, IFN-γ), Th2 cytokines (IL-4, IL-6 and IL-10), Macrophage-derived cytokine (IL- 12p40+p70), and Th17 cytokine (IL-17A)*

Monoclonal antibodies specific for different cytokines (IL-2, IFN-γ, IL-6, IL-12, and IL-17) were coated onto the wells of the microtiter strips provided during the first incubation. Then, IL-2, IFN-γ, IL-4, IL-6, IL-10, IL-12 and IL-17 present in the samples or standards were added into the perspective wells. Then a monoclonal antibodies against IL-2/IFN-γ/IL-4/IL-6/IL-10 and IL-17 conjugated to biotin were added into each well.Following incubation, unbound IL-2, IFN-γ, IL-4, IL-6, IL-10, IL-12, and IL-17 were removed during a wash step. Streptavidin-horse-raddish peroxidase (HRP) was added and bound to the biotyinylated anti-IL-2, IFN-γ, IL- 4, L-6, IL-10, IL-12 and IL-17. After incubation and wash step, a substrate solution reactive with HRP was added to the wells. A colored product was formed in proportion to the amounts of IL-2, IFN-γ, IL-4, IL-6, IL-10, IL-12 and IL-17 present in the sample. The reaction was terminated by addition of acid and the absorbance was measured at 450 nm. The limit of detection for IL-2, IFNγ, IL-4, IL-6, IL-10, IL-12 and IL-17 was 5.0 pg/mL, 2.0 pg/mL, 2.0 pg/mL, 2.0 pg/mL, 2.0 pg/mL, 2.0 pg/mL, and 50 ng/mL, respectively.

*Statistical analysis*

After reading the OD resulting obtained by the line equation and data normalization, levels of all parameters were calculated, and compared between two groups by the SPSS V16.0, using independent *t-*test and p-value < 0.05 was considered significant. 

## Results

Forty-seven subjects (23 PV patients, and 24 healthy controls) were studied. and 24 healthy controls) were studied. The mean ± SD of age was 40.19 ± 14.61 years (range, 17-80 years) in PV patients with a female to male ratio of 14:9, and 37.13 ± 17.67 (range 20-56 years) in the healthy controls. The demographic features of the patients have been summarised in Table 1.

*Th1 cytokine: IL-2*

The serum levels of IL-2 in most patients and controls were undetectable. Nevertheless, the mean serum levels of this cytokine in two patients was 85.82 pg/mL. 

*Th1 cytokine: IFNγ*

The serum levels of IFNγ in the patients and controls were undetectable.

*Th2 cytokine: IL-4*

The serum levels of IL-4 in most patients and controls were undetectable. Nevertheless, the mean serum levels of IL-4 in two patients was 194.55 pg/mL.

*Th2 cytokine: IL-10*

The serum levels of IL-10 in the patients and controls were undetectable, nevertheless, the mean serum levels of this cytokine was 64.375 pg/mL in four patients.

*Pro-inflammatory macrophage and T-cell derived-cytokine: IL-6*

The mean serum levels of IL-6 increased significantly in the patients, compared to the controls (169.50 vs. 75.62 pg/mL) (P < 0.05) (Figure 1).

*Pro-inflammatory Macrophage-derived cytokine (IL-12)*

The mean serum levels of IL-12 increased significantly in the patients, compared to the controls (135.33 vs. 86.28 pg/mL) (P < 0.05) (Figure 2).

*Th17 cytokine: IL-17*

The means serum levels of IL-17 in the patients and controls were 0.424 and 0.490 pg/mL, respectively. There was no significant difference between the serum levels of IL-17 in the patients and controls (P > 0.05). 

The results of Th1 and Th2 cytokines have been summarised in Table 2.

## Discussion

Systemic corticosteroids have been the mainstay of treatment for PV. An immunosuppressive agent has also been used in some patients as a primary agent or as a supplement to systemic corticosteroids. The undeniable side effects of these drugs lead to efforts for introducing new drugs for PV (16, 17). The present study provide evidence that the Th1 cytokine IL-2 was undetectable in the sera of patients and controls, with exception of two patients, who had the mean serum levels of 85.82 pg/mL. Demographic studies showed that PV has relapsed in these two patients, and this was one of the prominent findings in this study. This finding is consistent with the finding of Blitstein *et al*., who have found that an increase in the serum levels of IL-2 plays an essential role in the PV recurrence (18, 19). In addition, Prussick *et al*. have also found that IL-2 therapy has been connected with the recurrence of PV. Perhaps, this may be due to gradual increase of IL-2 following the infliction. The result of this gradual increase is the relapse of the disease (19, 20).

The current study also revealed undetectable serum levels of Th1 cytokine IFN-γ in the PV patients, nevertheless other studies among PV patients show variation in the serum levels of this cytokine (2, 21). The discrepancy between this study and other studies in the literature is unknown, but it may be due to sample size, genetic background, and methodology. 

Th2 cytokine IL-4 may confer the unanticipated data due to gene polymorphism (22, 23), and we found undetectable serum levels of IL-4 in most PV patients. Nevertheless, 

this study showed an increase in the serum concentrations of IL-4 in two PV patients (194.55 pg/mL). This finding is in agreement with the findings of D’Auria *et al*. who have described Th2 in PV (24). Thus, we may conclude that the high serum level of IL-4 may participate in the pathogenesis of PV, as IL-4 is involved in allergy and other immune system disorders. 

The current study also showed undetectable serum levels of Th2 cytokine IL-10. This finding is corroborated by the findings of Bhol *et al*., who have also found undetectable serum levels of IL-10 in the PV patients (25). This study also showed that the serum levels of IL-6 was elevated in the PV patients of PV confirmed by the histopathology and direct immunofluorescence method. IL-6 is a proinflammatory cytokine, and since PV is an inflammatory disease, therefore it is logical to conclude that the high level of IL-6 in the sera of these patients has been due to inflammatory nature of this disease (26).

Our data also revealed that the mean serum levels of IL-12 significantly increased in the sera of patients, compared to the controls. To the best of our own knowledge, there are no studies to measure the serum levels of this cytokine in the PV patients. Concerning the increased serum levels of IL-12, we argue that inhibition of the Th2 profile in most of our own patients (IL-4 and IL-10) may be caused by IL-12. Since the serum level of IL- 12 increased in PV patients, it may have been caused for declined serum levels of IL-4 and IL-10. However, it should have stimulated the Th1 cytokine IL-2 response, which was found to be high in two patients (27). 

The results showed that there was no significant difference between the serum levels of IL- 17 in the patients and controls. In addition, Arakawa *et al.*, have recently reported lesional Th17 cells in PV. The conflicting results in these studies may reflect the genetic variations (28). In conclusion, further investigations with larger numbers of patients with PV will be necessary to elucidate this discrepancy. 

Keskin et al. have suggested that both common immunosuppressive therapy and IV immunoglobulin therapies are analogous in their capacity to affect a group of cytokines in patients with PV (29). The isotype of antibodies generated by a known B cell is relied on the type of T helper lymphocytes (30). For example, T-cells which secrete Th1 cytokines, stimulate B cells to make IgG1, whereas Th2-type cytokines bring about B cells to secrete IgG4 (31, 32). Because IgG4 is the major isotype of the anti-Dsg1 and anti-Dsg-3 autoantibodies, it is thought that T-cells of the Th2 helper cell lineage may be related in PV (33). Circulating IgG auto-antibodies are present in a substantial percentage of PV patients in remission, because these patients have elevated levels of antibody production during active stages. The raised level of IL-6 (proinflammatory cytokine) and declined level of IL-2 (Th1 cytokine) in the current study supports this theory, because T-cell of the patients produce more IL-6, but not IL-2, suggesting that they secrete a Th2-like cytokine profile.

Present findings suggest the roles of Th1/Th2 type cytokines as well as macrophage-derived cytokine (IL-12) in the immunological disturbances in the pathophysiology of PV patients (2). These observations might suggest the potential clinical application of Th1/Th2 type cytokine-based therapy in PV.

## Conclusions

Based on the results of this study it can be concluded that the Th2-derived cytokine (IL-6) and macrophage-derived cytokine (IL-12) have essentioal roles in PV pathophysiology. In addtion the potential clinical application of Th1/Th2 type cytokine-based therapy in PV should be considered in next studies.
